# Supplementary material for: An unexpected N-dependence in the viscosity reduction in all-polymer nanocomposite
Source: Nat Commun. 2019 Dec 5;10:5552. doi: 10.1038/s41467-019-13410-z (PMC6895191; doi:10.1038/s41467-019-13410-z)
Supplement: Supplementary file 1 — Supplementary Information [file 41467_2019_13410_MOESM1_ESM.pdf]

Supplementary Materials for: An unexpected N-dependence in the viscosity reduction in all-polymer nanocomposite

Tao Chen,* Huan-Yu Zhao,* Rui Shi,* Wen-Feng Lin, Xiang-Meng Jia, Hu-Jun Qian,† and Zhong-Yuan Lu
State Key Laboratory of Supramolecular Structure and Materials, Institute of Theoretical Chemistry, Jilin University, Changchun 130023, China

Xing-Xing Zhang, Yan-Kai Li, and Zhao-Yan Sun
State Key Laboratory of Polymer Physics and Chemistry, Changchun Institute of Applied Chemistry, Chinese Academy of Sciences, Changchun, 130022, China

(Dated: November 1, 2019)

I. SUPPLEMENTARY METHODS

Simulation method and simulation details

Coarse-grained model and simulation details: Due to a large span in time and length scales in the system, a coarse-grained (CG) polystyrene model [1] is used in this study. In this model, each styrene unit is coarse-grained into one CG bead and the chain sterochemistry is well captured by maintaining different bond and angle types in the CG model. Each CG bead in simulation has a molar mass of a sytrene monomer, i.e., 104 g/mol. All interaction potentials, including nonboned and bonded interactions between CG beads in this model is in tabular form. All these interaction potential tables are available on the request to the authors. Based on this CG PS chain model, the model of the single-chain PS NP is prepared by a simulated intrachain cross-linking reaction process in dilute solution [2]. Internal structural properties of the linear PS chains and NPs can be well captured in our simulation as have been demonstrated in our previous works. [1, 2] Each NP in our simulation contains 250 monomers and 20% of them are cross-linking units. These NPs are indicated as NP250 in the following text. These NPs have an average radius of $\langle r_a \rangle =$ 2.0 nm, which is exactly the same as the value reported in experiment [3]. All the simulated systems are listed in Supplementary Table 1. The initial configurations of PNC systems are prepared by randomly generating NPs and melt chains in a cubic box with a density of 0.65 g/cm^3. Firstly, NPs prepared from the above mentioned simulated intrachain cross-linking reaction process are put on the randomly selected but relatively homogeneous positions in the simulation box, a preset minimum interparticle distance is used to avoid the direct contacts between these NPs. Afterward, the linear PS chains are generated by means of a continuous random walk in the box, where direct contacts between polymer chains and NPs are avoided. All simulations in this study are performed with GPU-accelerated GALAMOST simulation package. [4] To fully relax the system, all systems are subjected to an annealing process which can eventually result in a well equilibrated configuration at 500 K. The detailed preparation process of the cross-linked single-chain PS NP model and the annealing process for the equilibration of the initial configuration can be found in the following sections. The production run for each system is simulated up to 20 μs depends on the chain length and system size under isothermal-isobaric (NPT) condition at 1 atm and 500 K. For all the simulations, Berendsen thermostat (coupling time 0.5 ps) and barostat (coupling time 5 ps) are used to control the system temperature and pressure. The velocity-Verlet algorithm is used to integrate the Newtons' equations of motion in the system with a time step of $\delta t = 5$ ps. Generally it takes $\sim$ 60 days for 20 μs simulation paralleled on 4 NVIDIA GTX980 GPUs. Due to the same composition and therefore the same interaction parameter between NP and melt polymer chain monomers, NPs have a good dispersion in the system, as indicated by a representative snapshot in Supplementary Figure 1 during the final stage of the simulation. In general, the NPs are well dispersed in all the simulated systems.

Preparation of the cross-linked NP model in simulation: In experiment, cross-linked single-chain PS nanoparticles are synthesized in dilute solution by an internal cross-linking process, in which the cross-linker is benzocyclobutene (BCB). To faithfully simulate this process, we adopted following steps to generate the cross-linked NPs in our simulations: (1) A single chain is picked up from well-equilibrated melt and then this chain is put in solvent of PS2 dimer, which has a similar molecular size as phenyl ether used in experiments. (2) Then some styrene monomers along the PS chain are randomly chosen and assigned as cross-linkers. A cross-linking process in a 40 ns NVT simulation is performed, which is similar to the simulation of Liu, Mackay and Duxbury [5]. The distance between every possible pair of cross-linkers are checked every 0.8 ps. If the distance of two cross-linkers is in the range of (0.73 ± 0.1) nm, then a chemical bond will be created between them. Here 0.73 nm is the equilibrium distance between the centres of mass of two cross-linked BCB units measured from a well-optimized atomistic model. To ensure the cross-linking process is reasonable, two cross-linking reaction is only allowed between

* These authors contributed equally to this work
† Corresponding author, email: hjqian@jlu.edu.cn

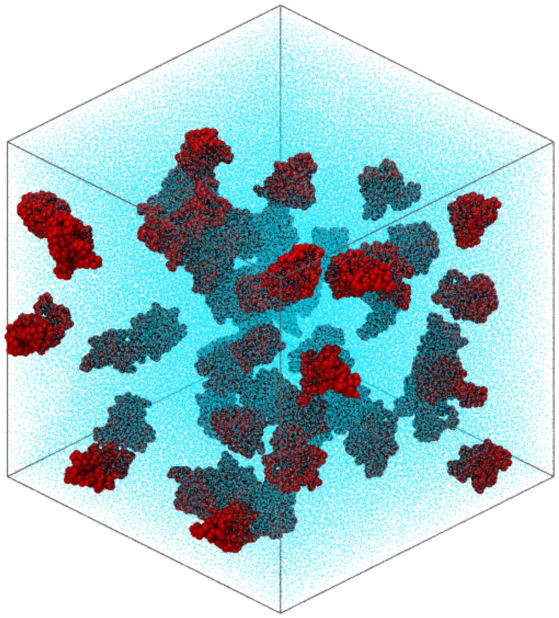

Supplementary Figure 1. Representative snapshot for system containing 10% NP250 in PS500 melt.

Supplementary Table 1. Simulated systems. N indicates the length of the melt PS chains. ϕ is the NP loading, L the box size, t the total simulation time and τ_d the disentanglement time obtained from Rouse mode analysis. For all the systems, there are totally $\sim 2.6\times10^5$ monomers in the simulation box.

N	n_{PS}	n_{NP}	ϕ (%)	L (nm)	τ_d (ns)	t (μs)
250	1040	0	0	35.5	135	1
250	1019	21	2	35.5	130	1
250	988	52	5	35.5	129	1
250	936	104	10	35.5	126	1
250	832	208	20	35.5	122	1
250	728	312	30	35.4	117	1
250	624	416	40	35.4	112	1
250	520	520	50	35.4	110	1
350	742	0	0	35.5	365	3
350	728	21	2	35.5	344	3
350	668	104	10	35.5	336	3
350	594	208	20	35.5	305	3
350	520	312	30	35.4	284	3
500	520	0	0	35.5	1059	5
500	510	21	2	35.5	1014	5
500	494	52	5	35.5	1009	5
500	468	104	10	35.5	922	5
500	416	208	20	35.5	891	5
500	364	312	30	35.4	769	5
500	312	416	40	35.4	776	5
500	260	520	50	35.4	657	5
720	361	0	0	35.5	3665	20
720	353	21	2	35.5	3225	20
720	325	104	10	35.5	3360	20
720	289	208	20	35.5	2569	20
720	253	312	30	35.4	2394	20
720	216	416	40	35.4	1896	20

BCB units which are at least separated by 14 monomers (about 2 Kuhn segments) along the chain backbone. Besides, since BCB unit has only two more carbon atoms than the benzene ring, we simply adopt the same nonbond CG potential as PS monomers in our simulation. A Gaussian potential [2] is used to describe the newly-formed bonds between cross-linkers so that the equilibrium bond length of cross-linking bond is 0.73 nm and the bond strength is similar to the normal PS bond used in our simulations.

Annealing process for the equilibration of the initial configuration: The initial configurations are generated at a density of 0.65 g/cm3. The cross-linked NPs are randomly placed in the simulation box without overlapping; the linear PS chains are then generated around the NP by a random walk process with a bond length of 0.5 nm, where direct contact polymer chains with NPs are avoided. A random series of R or S absolute configurations are specified with a half/half ratio on PS chains. To efficiently equilibrate the system and release the local stress, following annealing cycles are performed to equilibrate the system: (1) First, local packing and chemical bonds are relaxed using a softcore nonbonded interaction potential in a short NVT run of 2 ns with a small time step of 0.5 fs. During this process the full bond potentials are used to keep the connectivity of the polymer chain with the angle potentials switched off. (2) Second, angles are slowly relaxed by adding a soft-core angle potential in an NVT simulation of 0.2 ns, where the angle potential is replaced by a plateau when the potential energy is higher than 85.0 kJ/mol. (3) After the relaxation of local packing and local bonds/angles, we switch on all the full potentials and slowly enlarge the time step δt from 0.5 to 5 fs in sequentially performed NVT simulations: an NVT run for 0.1 ns with $\delta t = 0.5$ fs followed by another 0.1 ns with $\delta t = 1$ fs, then for 0.5 ns with $\delta t = 2$ fs, and for 25 ns with $\delta t = 5$ fs. Finally, the system is equilibrated under NPT condition for 25 ns with $\delta t = 5$ fs. All above equilibration runs are performed at a high temperature of T = 1000 K. More details are completely described in our previous work [2]. After the relaxation at high temperature described above, the system is addressed to an annealing process being repeatedly performed between 1000 and 500 K. Namely, the system is first slowly cooled down from 1000 to 500 K and then slowly heated back up to 1000 K. Afterward, this cooling and heating process is repeated once again and finally the system is cooled down to 500 K. The step size of the temperature change in this process is always ± 50 K. For instance, the cooling process is performed gradually from 1000 K to 950, 900, 850, ..., 550, and finally 500 K. The simulation on each temperature is composed of 1 ns NVT simulation followed by 2 ns NPT simulation with an atmospheric pressure. After this equilibration process, the system configuration can be well equilibrated.

Rouse mode Analysis: For a polymer chain with N

monomers, the Rouse modes are defined as:

$$\mathbf{X}_p \equiv \frac{1}{N}\sum_{i=1}^{N}\cos[\frac{p\pi}{N}(i-\frac{1}{2})]\mathbf{r}_i(t) \qquad (1)$$

where p is the mode index and $\mathbf{r}_i(t)$ is the tracjectory of ith monomer [6–8]. The $p = 0$ mode describes the motion of the center of mass of the chain, while the other modes describe the relaxation of segments with N/p monomers. For an ideal chain, the Rouse modes are the normal modes and they relax exponentially:

$$\langle \mathbf{X}_p(t)\cdot\mathbf{X}_p(0)\rangle/\langle\mathbf{X}_p\rangle = \exp[-(t/\tau_p)] \qquad (2)$$

However, for real polymer chains including those widely used in computer simulations, the Rouse modes relaxation often follows a stretched exponential function [6–8]:

$$\langle \mathbf{X}_p(t)\cdot\mathbf{X}_p(0)\rangle/\langle\mathbf{X}_p\rangle = \exp[-(t/\tau_p)^{\beta_p}] \qquad (3)$$

To better describe the relaxation of the pth mode, an effective relaxation time is defined as:

$$\tau_p^{\text{eff}} = \int_0^\infty \exp[-(t/\tau_p)^{\beta_p}]\,dt = (\tau_p/\beta_p)\,\Gamma(1/\beta_p), \qquad (4)$$

where $\Gamma(x)$ is the Gamma function. With this equation, a series of τ_p^{eff} that describe the relaxation process at different length and time scales can be obtained.

Alternatively, effective chain relaxation time τ^{eff} is also obtained in our simulation via calculating the auto correlation function of chain end-to-end vector $R(t)$ as follows:

$$< R(t)R(0) >= \exp[-(t/\tau)^{\beta}], \qquad (5)$$

$$\tau^{\text{eff}} = \int_0^\infty \exp[-(t/\tau)^{\beta}]\,dt = (\tau/\beta)\,\Gamma(1/\beta), \qquad (6)$$

Analysis of primitive path: The topological confinement effect of polymer chains is analyzed by using Z1 code [7, 9–11] for our simulated systems. Here we use the same approach as described in Ref. 7. The so-called phantom particle limit is adopted, i.e., the NPs are removed during the analysis of primitive path, and the $\langle \text{Z}_{\text{kink}}\rangle$ is used as the variable to describe the entanglement. The Z1 analysis is performed every 50 ns for the trajectory of all systems.

Experimental details

Materials: Alkoxyamine initiator, N-tert-Butyl-N-(2-methyl-1-phenylpropyl)-O-(1-phenylethyl) hydroxylamine, is purchased from Sigma-Aldrich, 4-vinylbenzocyclobutene is purchased from EFEBIO, Shanghai, China. Benzyl ether is purchased from Sigma-Aldrich and the moisture and impurities are removed with sodium reflux. The final solvent is obtained by distillation under reduced pressure. Other commercial reagents are obtained from Sinopharm Chemical Reagent Co., Ltd and used without further purification. Linear polystyrene (PS) is purchased from Polymer Source Inc. at molecular weights of 5.5, 98, and 202 kDa, they are further purified via a reprecipitation process before use. Details of these polymers are given in Supplementary Table 5.

Supplementary Table 2. The calculated $<R_{\text{g}}>^{1/2}$ and $<R_{\text{ee}}>^{1/2}$ values (in unit of nm) of PS chains in PS500/NP250 and PS720/NP250 systems.

PS500/NP250	0%	5%	10%	20%	30%	40%	50%
$<R_{\text{g}}>^{1/2}$	5.74	5.72	5.71	5.71	5.66	5.62	5.51
$<R_{\text{ee}}>^{1/2}$	14.06	14.03	14.02	13.96	13.91	13.75	13.47
PS720/NP250	0%	2%	10%	20%	30%	40%	
$<R_{\text{g}}>^{1/2}$	6.93	6.95	6.96	6.86	6.74	6.73	
$<R_{\text{ee}}>^{1/2}$	17.00	17.13	17.10	16.75	16.50	16.47	

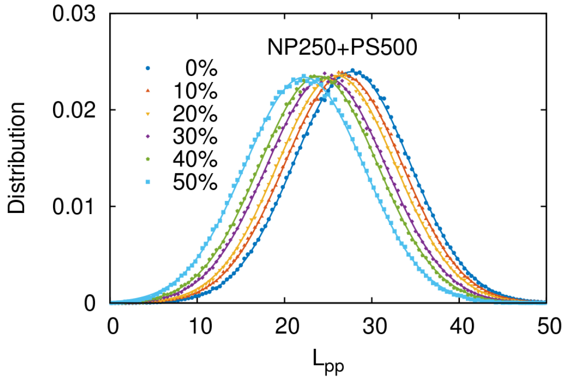

Supplementary Figure 2. The distribution of $< L_{\text{pp}} >$ for PS500 chains in systems containing different loadings of NP250. The solid lines are fitted with Gaussian.

General Methods: Nuclear magnetic resonance is performed on a Bruker AVANCE 400 FT-NMR spectrometer using deuterated solvents and the solvent peak as a reference. Gel permeation chromatography is performed in tetrahydrofuran (THF) on an Agilent 1260 Infinity chromatograph equipped with an Agilent column (PLgel 5- m Mixed-D, 300mm × 7.5 mm). An Agilent G1362A refractive index detector (RID) was employed. The polystyrene molecular weights are calculated relative to linear polystyrene standards. Dynamic Light Scattering measurement are carried out on a Malvem Zetasizer Nano ZEN3600.

Synthesis and Characterization: The nanoparticles (NPs) were synthesized via intramolecular cross-linking reactions in ultra-dilute solution, details of the process are conducted according to Ref. [12].

Random Copolymer of Styrene and 4-vinylbenzocyclobutene: The alkoxyamine initiator, N-tert-Butyl-N-(2-methyl-1-phenylpropyl)-O-(1-phenylethyl)hydroxylamine, (42 mg, 0.129 mmol),

Supplementary Table 3. The average length of the primitive path $<L_{\rm pp}>$ and its standard deviation $\sigma_{L_{\rm pp}}$ in pure polymer system and composite system with a NP loading of 20%.

	PS250/NP250		PS350/NP250		PS500/NP250		PS720/NP250	
NP loading (%)	0%	20%	0%	20%	0%	20%	0%	20%
$<L_{\rm pp}>$	14.98	14.06	19.99	18.72	27.70	26.74	38.73	36.37
$\sigma_{L_{\rm pp}}$	4.65	4.56	5.54	5.54	6.66	6.76	8.14	8.36

Supplementary Table 4. The number of entanglements per chain ($<Z_{\rm kink}>$) and its standard deviation ($<\sigma_Z>$), and the relaxation time ($\tau^{\rm eff}$) of melt polymer chains plotted in Figure 6 in the main text.

NP loading (%)	$<Z_{\rm kink}>$	$<\sigma_Z>$	$\tau^{\rm eff}$
PS500/NP250			
0	6.11261	0.158101	1.05936e+06
5	5.84303	0.162863	1.0095e+06
10	5.67919	0.152475	922971
20	5.2252	0.128084	891020
30	4.7485	0.140937	769618
40	4.33691	0.127831	776068
50	3.75906	0.158927	657013
PS720/NP250			
0	8.53255	0.238132	3.66561e+06
2	8.53129	0.220715	3.22532e+06
10	8.10972	0.197967	3.3604e+06
20	7.4358	0.167593	2.56989e+06
30	6.76912	0.22526	2.39463e+06
40	6.231	0.21533	1.89656e+06

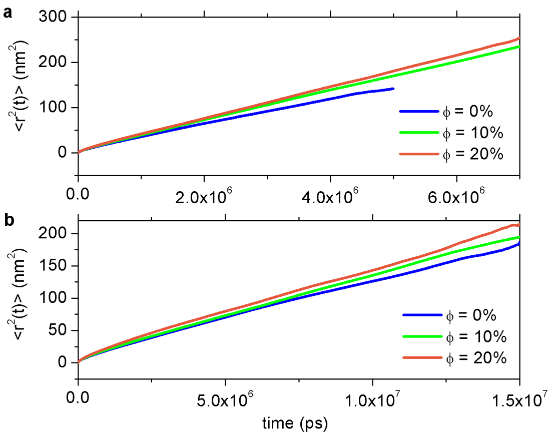

Supplementary Figure 3. Results of MSD for the center of mass of the melt linear PS chains in systems with different NP loadings, ϕ = 0% (blue), 10% (green) and 20% (red). They are the same data as in Figure 2 of the main text. MSD of NP is not shown here for clarity. (a) is for the composite PS500/NP250, and (b) is for the composite PS1300/NP250.

dissolved in styrene (3.749 g, 36.0 mol) and 4-vinylbenzocyclobutene, (1.175 g, 9.0 mmol), is added to a glass ampule with a stir bar. After three freeze and thaw cycles the ampule is sealed under nitrogen and heated for 6 h at 120 °C. The resulting polymer is dissolved in dichloromethane and purified by precipitation into a 1:1 mixture of 2-propanol/acetone followed by reprecipitation into methanol to give product as a colorless powder, Mw = 26200; PDI = 1.08; 1H NMR (500 MHz, $CDCl_3$) δ 7.24-6.57 (m, ArH), 3.05 (br s, CH_2), 1.83-1.26 (m, CH_2, CH).

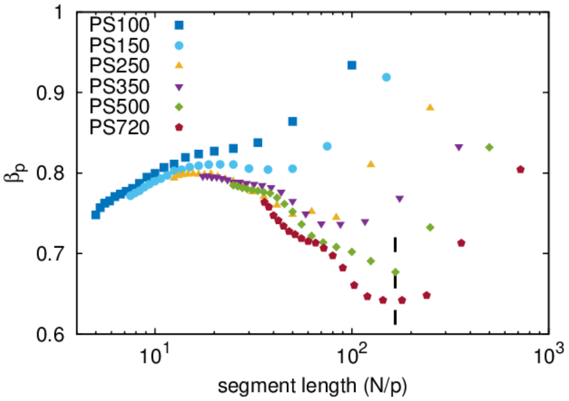

Supplementary Figure 4. The $\beta_{\rm p}$ values plotted as a function of segement length (N/p) for the first 20 modes of pure PS chains with different lengths. The minimum of β is located at $N/p \sim 166$ (indicated by a vertical black dash line), which is close to entanglement length $N_{\rm e}$.

Supplementary Table 5. Linear polystyrene used in this study.

Sample	M_w (kDa)[a]	M_n (kDa)[a]	PDI
PS202kDa	201806	185356	1.0887
PS98kDa	98461	81039	1.2150
PS54kDa	54627	49455	1.1046

[a] Mw is the weight-averaged molecular weight and Mn is the number-averaged molecular weight. The molecular weights for the linear chains are determined by GPC.

General Procedure for the Nanoparticle Synthesis: In a 1000-mL three-necked flask equipped with a internal thermometer, condenser, and septum, 180 mL of benzyl ether is heated at 250 °C under argon. A solution of the benzocyclobutene (BCB)-functionalized linear polymer, (0.70 g, Mn =26200; PDI = 1.08; 20mol % BCB), dissolved in benzyl ether (60 mL) is added dropwisely via a peristaltic pump at ca. 200 L/min with vigorously stirring under nitrogen. After addition the reaction mixture is heated for an additional 1 h, the remaining crude product is dissolved in dichloromethane and precipitated into methanol. The above procedure

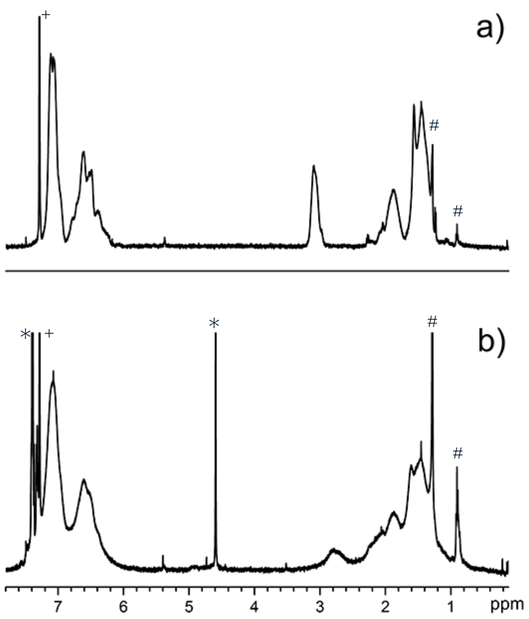

Supplementary Figure 5. Comparison of ^{1}H NMR spectrum (500 MHz, 298 K) for (a) the linear precursor random block copolymer chain and (b) the resulting nanoparticle. Solvent peak were marked by (+) d-Chloroform, ($\star$) benzyl ether, (#) n-Hexane.

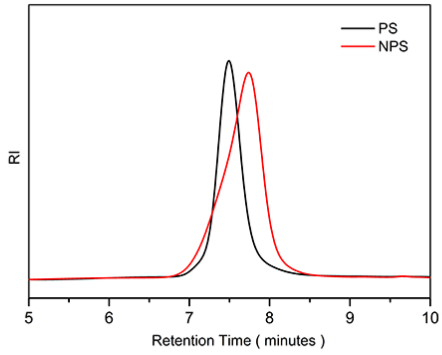

Supplementary Figure 6. GPC trace overlay.

generates nanoparticles as a colorless solid. Supplementary Figure 5 shows the results of ^{1}H NMR (500 MHz, $CDCl_3$). The significant change is the disappearance of the aliphatic benzocyclobutene protons at 3.05 ppm due to the formation of the cross-linked nanoparticles; all other aspects of the spectrum are similar. Results of GPC and DLS data shown in Supplementary Figure 6 and Supplementary Table 6 indicate an apparent contraction of the NP compared with the precursor random block copolymer chain after the intra-chain cross-linking reaction.

Supplementary Table 6. DLS and GPC data for precursor random block copolymer chains and corresponding cross-linked nanoparticles.

Sample	R_h (nm)[a]	M_n (kDa)[b]	M_w (kDa)[b]	PDI
precursor chain	5.09	24.2	26.2	1.0811
nanoparticle	4.16	18.7	22.0	1.1745

[a] R_h is the hydrodynamic radius determined by Dynamic Light Scattering method;
[b] M_n is weight-averaged molecular weight and M_n is the number-averaged molecular weight, they are determined by GPC for both linear precursor chains and cross-linked nanoparticles.

Supplementary Table 7. Zero-shear viscosity of pure linear PS melts and composite systems containing 2% 25 kDa NPs at T = 170 °C.

M_w (kDa)	linear PS	composite
54.6	2132.6	1825
98.5	17211.7	12306
201.8	233105	102182

Rheology Measurements: Blends of the PS nanoparticles with linear PS are prepared through co-dissolution in dichloromethane and drop on the PEI membrane to form thin film, followed by drying in a vacuum at 80 °C for at least a week to ensure complete solvent removal. The 25 mm aluminium diameter disks are placed on the 25 mm aluminium parallel plates fixture of a Discovery Hybrid Rheometer set at a gap of approximately 1 mm. Measurements were performed in the oscillatory frequency mode at various temperatures, and then combined by time-temperature superposition to generate a master curve at 170 °C. The strain was set small enough so that all response was in the linear viscoelastic region. To ensure the sample arrived target temperature, all samples spent 10∼15 min before commencing measurements. Supplementary Table 7 lists the zero-shear viscosity data for both pure linear PS melts and composite systems.

SUPPLEMENTARY REFERENCES

[1] Hu-Jun Qian, Paola Carbone, Xiaoyu Chen, Hossein Ali Karimi-Varzaneh, Chee Chin Liew, and Florian Müller-Plathe, "Temperature-transferable coarse-grained potentials for ethylbenzene, polystyrene, and their mixtures," Macromolecules **41**, 9919 (2008).

[2] Tao Chen, Hu-Jun Qian, You-Liang Zhu, and Zhong-

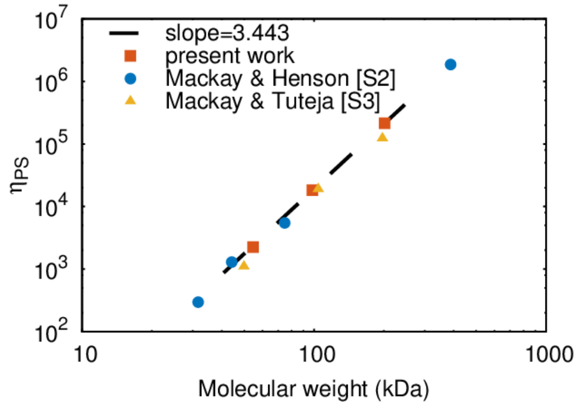

Supplementary Figure 7. Zero-shear viscosity of pure linear PS melts as a function of molecular weight at 170 °C. Results from other works (refs. [13, 14]) are also plotted for comparison. It shows a very good agreement.

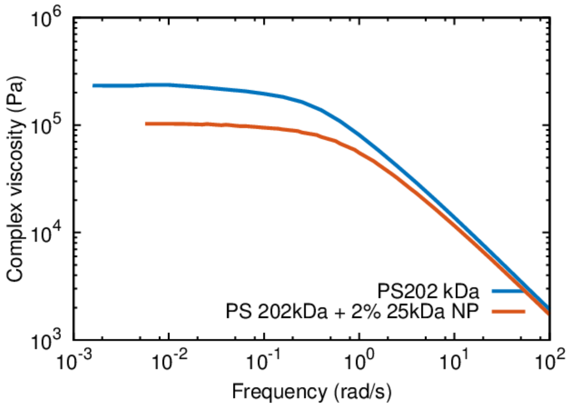

Supplementary Figure 8. The representative results of the complex shear viscosity as a function of frequency for both pure melt with a molecular weight of 202 kDa for linear PS chains and its composite with a NP loading of 2%. The NPs have a molecular weight of 25 kDa.

Yuan Lu, "Structure and dynamics properties at interphase region in the composite of polystyrene and cross-linked polystyrene soft nanoparticle," Macromolecules **48**, 2751 (2015).

[3] Anish Tuteja, Phillip M. Duxbury, and Michael E. Mackay, "Polymer chain swelling induced by dispersed nanoparticles," Phys. Rev. Lett. **100**, 077801 (2008).

[4] You-Liang Zhu, Hong Liu, Zhan-Wei Li, Hu-Jun Qian, Giuseppe Milano, and Zhong-Yuan Lu, "Galamost: Gpu-accelerated large-scale molecular simulation toolkit," J. Comput. Chem. **34**, 2197 (2013).

[5] J. W. Liu, M. E. Mackay, and P. M. Duxbury, "Molecular dynamics simulation of intramolecular cross-linking of bcb/styrene copolymers," Macromolecules **42**, 8534–8542 (2009).

[6] Jagannathan T. Kalathi, Sanat K. Kumar, Michael Rubinstein, and Gary S. Grest, "Rouse mode analysis of chain relaxation in homopolymer melts," Macromolecules **47**, 6925 (2014).

[7] Ying Li, Martin Kröger, and Wing Kam Liu, "Nanoparticle effect on the dynamics of polymer chains and their entanglement network," Phys. Rev. Lett. **109**, 118001 (2012).

[8] J. T. Padding and W. J. Briels, "Time and length scales of polymer melts studied by coarse-grained molecular dynamics simulations," J. Chem. Phys. **117**, 925 (2002).

[9] Nikos Ch Karayiannis and Martin Kröger, Int. J. Mol. Sci. **10**, 5054 (2009).

[10] Martin Kröger, Comput. Phys. Commun. **168**, 209 (2005).

[11] Sachin Shanbhag and Martin Kröger, Macromolecules **40**, 2897 (2007).

[12] Eva Harth, Brooke Van Horn, Victor Y. Lee, David S. Germack, Chad P. Gonzales, Robert D. Miller, and Craig J. Hawker, "A facile approach to architecturally defined nanoparticles via intramolecular chain collapse," J. Am. Chem. Soc. **124**, 8653 (2002).

[13] Michael E. Mackay and David J. Henson, "The effect of molecular mass and temperature on the slip of polystyrene melts at low stress levels," J. Rheol. **42**, 1505 (1998).

[14] A. Tuteja, M. E. Mackay, C. J. Hawker, and B. Van Horn, "Effect of ideal, organic nanoparticles on the flow properties of linear polymers: Non-einstein-like behavior," Macromulecules **38**, 8000 (005).
